# Multi-Kinase Inhibitor C1 Triggers Mitotic Catastrophe of Glioma Stem Cells Mainly through MELK Kinase Inhibition

**DOI:** 10.1371/journal.pone.0092546

**Published:** 2014-04-16

**Authors:** Mutsuko Minata, Chunyu Gu, Kaushal Joshi, Mariko Nakano-Okuno, Christopher Hong, Chi-Hung Nguyen, Harley I. Kornblum, Annie Molla, Ichiro Nakano

**Affiliations:** 1 Department of Neurological Surgery, The Ohio State University Medical Center, Columbus, Ohio, United States of America; 2 Department of Internal Medicine, The Ohio State University Medical Center, Columbus, Ohio, United States of America; 3 James Comprehensive Cancer Center, The Ohio State University Medical Center, Columbus, Ohio, United States of America; 4 Pharmaco-chemistry, UMR 176 CNRS-Institut Curie, Orsay, France; 5 Departments of Psychiatry and Molecular and Medical Pharmacology, University of California Los Angeles, Los Angeles, California, United States of America; 6 Institut Albert Bonniot, Université Joseph Fourier, Grenoble Cedex 9, France; 7 Departments of Neurosurgery, Beijing Sanbo Brain Hospital, Capital Medical University, Beijing, China; Cleveland Clinic, United States of America

## Abstract

Glioblastoma multiforme (GBM) is a highly lethal brain tumor. Due to resistance to current therapies, patient prognosis remains poor and development of novel and effective GBM therapy is crucial. Glioma stem cells (GSCs) have gained attention as a therapeutic target in GBM due to their relative resistance to current therapies and potent tumor-initiating ability. Previously, we identified that the mitotic kinase maternal embryonic leucine-zipper kinase (MELK) is highly expressed in GBM tissues, specifically in GSCs, and its expression is inversely correlated with the post-surgical survival period of GBM patients. In addition, patient-derived GSCs depend on MELK for their survival and growth both *in vitro* and *in vivo*. Here, we demonstrate evidence that the role of MELK in the GSC survival is specifically dependent on its kinase activity. With *in silico* structure-based analysis for protein-compound interaction, we identified the small molecule Compound 1 (C1) is predicted to bind to the kinase-active site of MELK protein. Elimination of MELK kinase activity was confirmed by *in vitro* kinase assay in nano-molar concentrations. When patient-derived GSCs were treated with C1, they underwent mitotic arrest and subsequent cellular apoptosis *in vitro,* a phenotype identical to that observed with shRNA-mediated MELK knockdown. In addition, C1 treatment strongly induced tumor cell apoptosis in slice cultures of GBM surgical specimens and attenuated growth of mouse intracranial tumors derived from GSCs in a dose-dependent manner. Lastly, C1 treatment sensitizes GSCs to radiation treatment. Collectively, these data indicate that targeting MELK kinase activity is a promising approach to attenuate GBM growth by eliminating GSCs in tumors.

## Introduction

Glioblastoma multiforme (GBM) is the most common and lethal primary brain tumor in adults, and therefore, there is an urgent need to develop novel therapeutic strategies that effectively target therapy-resistant GBM cells. Among heterogeneous GBM cells glioma stem cells (GSCs) represent a subpopulation of highly tumorigenic cells that possess stem cell characteristics. While our understanding of GSCs is evolving, there is a great deal of evidence supporting the hypothesis that GSCs drive GBM propagation and promote resistance to conventional therapies such as radiation and chemotherapy [1]–[9].

Maternal embryonic leucine zipper kinase (MELK) is a serine/threonine kinase that is highly expressed in various organ-specific stem cells and cancers [10], [11]. Furthermore, MELK expression is correlated with a poor prognosis of a variety of cancers, including GBM [10]–[13]. We previously demonstrated that MELK is abundantly expressed in GBM with preferential expression in GSCs and that targeting MELK-mediated pathways disrupt cell cycle progression of GSCs *in vitro* and tumor growth *in vivo*, suggesting that MELK is a clinically relevant molecular target for GBM therapy [10], [14]–[17]. To gain insights in the mechanisms of action, we recently identified that MELK forms a protein complex with the oncogenic transcription factors c-JUN and FOXM1 in GSCs but not in non-GSCs or normal stem/progenitor cells [18], [19]. Further, both of these protein interactions are specifically dependent on the MELK kinase domain [18]. These results suggest that inhibition of the kinase activity of MELK could disrupt key interactions with pivotal oncogenes in cancer cells, while relatively sparing normal cells. In this study, we sought to identify a novel small molecule that potently inhibits MELK kinase activity.

## Materials and Methods

### Ethics

Experiments using de-identified human tissue-derived materials were carried out under the approved Institutional Review Board at University of California, Los Angeles (UCLA) or Ohio State University (OSU). Microarray studies were carried out at UCLA. Primary samples collected at UCLA were de-identified and sent to OSU for further studies. The OSU Institutional Review Board approved this research study and waived the need for further written informed consent from the participants. The name of this protocol is Investigating Novel Therapeutic Strategies for Brain Tumor Treatment and the approval number at OSU is 2005C0075. I Nakano serves as the Principal Investigator for this approved protocol. All animal experimentation was performed at OSU with the approval of the OSU Animal Research Committee, following NIH guidelines.

### Tissue Culture

Cells derived from 3 samples of GBM surgical tissues were established in Dr. Harley Kornblum’s laboratory at UCLA and were cultured as previously described [20]. Neurosphere cultures derived from these 3 samples were designated as GBM146, GBM157 and GBM206. GBM1600 cells were kindly provided by Dr. Paul Mischel at UCLA and cultured in DMEM/F12 with 10% fetal bovine serum (FBS) (Sigma-Aldrich, MO)[16]. U87 and U251 were obtained from ATCC (VA) and maintained in DMEM (Life technologies, NY) with 10% FBS (Life technologies, NY).

### cDNA Microarray

RNA was extracted from GBM sphere samples (GBM146, GBM157, and GBM206) treated with 1 μM Siomycin A or control (DMSO) for 24 hours with RNeasy Mini Kit according to the manufactur’s protocol (Qiagen). RNA samples were subjected to cluster (A) and canonical pathway analyses (D) by Ingenuity software (Ingenuity Systems, www.ingenuity.com). The GEO submission number for this microarray is GSE50227.

### Flow Cytometry and Cell Sorting

Flow cytometry and cell sorting of CD133(+) and CD133(−) cells from GBM spheres were performed using CD133 antibody (clone: AC133) according to manufacturer’s protocol (Miltenyi Biotec, CA) and as described previously [16], [17].

### *In silico* Docking Model and In vitro Kinase Assay

Using the structure-based virtual screening method, C1 was identified as a potential MELK inhibitor from readily available half a million commercial compounds. C1 compound was subsequently validated via experimental enzyme assay as previously described [21], [22]. Briefly, in order to account for the correct binding of the ligands, crystal structural complexes of kinases for each MELK inhibitor in the Protein Data Bank were sought and similarity analysis based on SMILES was carried through the National Center for Biotechnology Information. In total, 16 groups of templates, 4 structurally distinct MELK, were selected for induced fit MELK conformational modeling on the basis of backbone root-mean-square deviation of the binding site residues, visual inspection of the p-loop, and individual docking performance. Furthermore, the templates were selected using a MELK domain sequence with the Basic Local Alignment Search Tool. Using these conformers and known inhibitors, all the docking and virtual screening calculations were performed with the Virtual Screening Workflow script and selected the final 3 protein models. A total of 30 compounds of varying potency with at least 25% inhibition of MELK activity at 1 μM were collected and downloaded the structures from readily available half a million commercial compounds. With the screening to the ATP binding pocket of all 3 selected conformers using Glide HTVS docking, the top 10% of the compounds were carried forward by the more exhaustive Glide SP docking algorithm. The most highly scored 10% of the SP docked compounds were narrowed down and finally the 3 compounds, showed a pair of hydrogen bonds with the hinge residues, were selected. Subsequently, the 3 compounds were validated via experimental enzyme assays, C1 was the most selective (K_d_ = 18 μM), which showed no or minimal activity to the other kinases. Similarity search to the Chemical Abstracts Service database was performed in order to check the novelty of this computationally discovered MELK inhibitor candidate.

### GBM Slice Culture

GBM surgical tissues of 2 patients were received immediately after surgery from the Department of Pathology at OSU and they were histopathologically diagnosed as GBM by the assigned neuro-pathologists. Serial sections of the surgical specimens were cut to create tumor blocks (10 mm in diameter) and these blocks were transferred into 6 well plates as described previously [23]. Tumor blocks were then injected with either DMSO (5%) or C1 (2.5 nM) and incubated for 16 hours at 37°C in humidified air containing 5% CO_2_. After incubation we confirmed that the tumor slice cultures retain the histopathological characteristics of GBM. These treated tissues were fixed with 10 mL of 10% v/v formalin for 24 hours and processed for paraffin-embedded sections (4 μm thickness) for immunohistochemistry.

### Xenograft

Ten thousand GBM157 sphere cells in 5 μl of phosphate buffered saline (PBS) were injected intracranially into immunocompromised mice (n = 16) (Athymic NCr-nu/nu; National Cancer Institute, Strain Code 01B74) according to the methods described previously [19], [23]. At day 7 after transplantation, varying doses of C1 (2.5 pmol: n = 3 25 pmol: n = 4, 250 pmol: n = 5) or DMSO (n = 4) were injected into tumor cavities. Three days following C1 or DMSO injection, we sacrificed 3 treated mice (DMSO: n = 1, 25 pmol: n = 1, 250 pmol: n = 1) and stained the brains with the proliferation marker Ki-67. For analysis of tumor growth, 13 mice were sacrificed at 8 weeks after transplantation. The tissues were fixed with ice cold 4% paraformaldehyde in PBS overnight, sunk in 20% sucrose in PBS, and stored at −80°C until use. Sections were subsequently cryoprotected, sectioned at 20 μm and stained with the human-specific Nestin for measuring the tumor size according with the same protocol that we used in our previous study [16], [25].

### Immunocytochemistry and Immunohistochemistory

Studies were performed as described previously [16], [17], [19]. The primary antibody for MELK (1∶200, Sigma-Aldrich, Missouri) was used to visualize the fluorescent signals using the following secondary antibodies: Alexa 488 or Alexa 555 (1∶1000, Cell Signaling Technology, MA). Specificity was determined using no-primary control slides. For immunohistochemistry, the following primary antibodies were used: Nestin (anti-Nestin, clone 10C2, 1∶200, mouse monoclonal antibody, MAB5326, MA) and Ki67 (anti-Human Ki-67, clone MIB-1, 1∶1, mouse monoclonal antibody, Dako, Denmark). The Envision system (Dako) followed by Diaminobenzidine (DAB) method was used for detection of primary antibody according the manufacturer’s protocol. For paraffin-embedded slides, hematoxylin was used as a nuclear counterstain. IHC scoring was performed using automated digital image analysis (ImageJ).

### Time-lapsed Microscopy

U251 cells were transfected with the vector E-GFP-N1 using lipofectamine (Invitrogen) according to the manufacturer’s protocol. Cells were selected by gentamicin (100 μg/ml), seeded on a 2-wells Lab-Tek chambered coverglass (Nalge Nunc International), and maintained under standard culture conditions (37°C, 5% CO_2_) for 22 to 24 hours. One *μ*M of C1 or DMSO was added to the cell culture just before imaging. To avoid drug combinations, cells were only synchronized by trypsination around 24 hours before imaging. Images were acquired on a Zeiss dynascope confocal microscope (LSM 710) equipped for alive cells (37°C, 5% CO_2_) by using a Plan-Apochromat 40 X-water immersion objective. The positions of the mitotic cells on the stage were registered and therefore, these cells were imaged continuously. None of the followed mitotic cells divided in two daughter cells. Control and treated cells were imaged simultaneously. Then, the Lab-Teck was kept under normal culture conditions and cells were fixed upon 72 hours of treatment by 4% paraformaldehyde and nuclei stained by Hoechst 3342. Images were recorded with the same equipment as above but at room temperature and analyzed with the Zen software provided by Zeiss. Only one cell line was imaged since the behavior of the cells upon C1 treatment is similar to what was previously observed in HeLa, Hek or H358 cells [26], [27]. Three independent experiments were conducted and 10 to 15 fields were followed in each.

### Apoptosis Assay

U251 cells treated with either C1 or DMSO for 48 hours was analyzed by flow cytometry with Annexin V antibody and Propidium Iodide (Life technologies, NY) using the Apoptosis Detection Kit (R&D Systems, MN) according to the manufacturer’s instructions. Data was confirmed by 3 independent experiments.

### Neurosphere Formation Assay and Radiosensitivity Assay

Neurosphere formation assay and radiosensitivity assay were performed as described previously [16], [17], [19].

### Statistical Analysis

Statistical analysis was performed using the SPSS17 Statistics software (IBM Corporation, NY) using one-way ANOVA and student’s T test. A probability of p<0.05 was considered to be significant. All the data are shown in mean ± standard error of the mean (SEM).

## Results

### Siomycin a Treatment of GSCs Results in Downregulation of Genes in the DNA Damage-induced Repair Pathway

**Figure 1:**
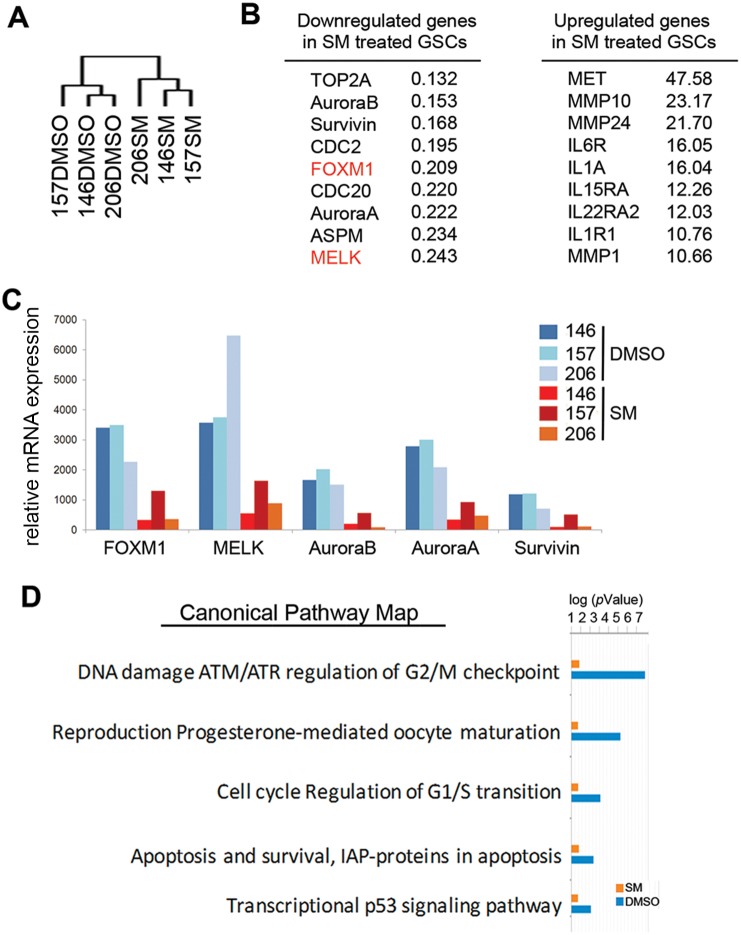
Genes in the DNA damage-induced response pathway are downregulated in Siomycin A-treated GSCs. cDNA microarray of GBM146, GBM157, and GBM206 samples treated with 1 μM Siomycin A or control (DMSO) were subjected to cluster (A) and canonical pathway analyses (D) using Ingenuity software. Log (*p*Value) of most significantly downregulated pathways are shown (*p*<0.05). The most downregulated and upregulated genes in Siomycin A-treated GSCs are shown in (B) and (C), respectively. Expression of FOXM1, MELK, Aurora A/B, and Survivin were significantly decreased by Siomycin A treatment compared with DMSO treatment.

Previously we demonstrated that the thiazole antibiotic Siomycin A attenuates a MELK-mediated signaling, thereby diminishing GSC growth *in vitro* and *in vivo* [16]. Here we first sought to determine the downstream pathways in GSCs that are suppressed by Siomycin A treatment. We performed cDNA microarray with 3 well-characterized GBM neurosphere samples (GBM146, GBM157, and GBM206) [10] treated with either 1 μM of Siomycin A or vehicle (DMSO) for 48 hours. Unbiased cluster analysis separated these 3 samples into 2 groups; either DMSO-treated or Siomycin A-treated GBM neurospheres (Fig. 1A). Consistent with our previously published quantitative reverse transcriptase (qRT)-PCR [19], Siomycin A significantly downregulated MELK as well as its binding partner FOXM1 (Fig. 1B). The other downregulated genes included mitotic genes such as Aurora A/B and Survivin (Fig. 1B and C). Pathway analysis using Ingenuity indicated that the most downregulated pathway is the DNA damage-induced ATM/ATR pathway; a pathway that regulates the G2/M checkpoint (Fig. 1D). In addition, the transcriptional p53 signaling pathway was also significantly downregulated by Siomycin A treatment (Fig. 1D), supporting our previous study that demonstrated MELK action is mediated, at least in part, through inhibition of p53 pathway [18].

### Kinase Activity of MELK is Essential for GSC Survival

**Figure 2:**
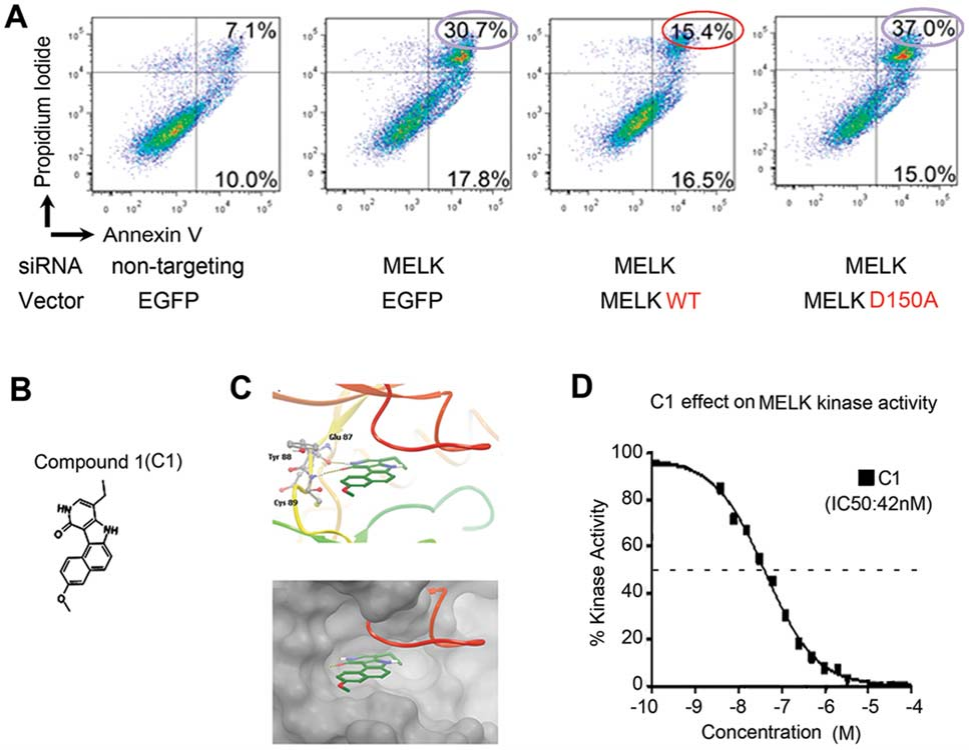
The kinase activity plays an essential role in the action of MELK on GSCs and identification of C1 as a MELK kinase inhibitor. A, GBM1600 spheres transfected with MELK wild type vector or MELK kinase dead (MELK D150A) vector were subjected to transfection of shRNA for MELK to eliminate endogenous MELK. Non-targeting shRNA and GFP vector were used as controls. The proportion of apoptosis at 48 hours post-transfection was analyzed by flow cytometry using Annexin V antibody and Propidium Iodide. B, Schema showing the chemical structure of the benzo[e]pyridoindole, Compound 1 (C1). C, *In silico* modeling of C1 predicted binding to MELK through hydrogen bonding with the backbone residues of the hinge region of the ATP binding pocket (Glu87, Tyr88, Cys89) by computational analysis. A pair of hinge residue hydrogen bonds makes favorable hydrophobic interactions. D, A ten-dose curve allows the determination of the *in vitro* efficiency of C1 towards the recombinant kinase (IC50: concentration of C1 that inhibits kinase activity by 50%). Solid black squares indicate individual data points.

Next we investigated whether the function of MELK in GSCs specifically depends on its kinase activity. To address this question, we combined lentiviral infection of MELK shRNA vector, to downregulate endogenous MELK protein, with overexpression of MELK wild type or the kinase dead version of MELK (MELK D150A) [28]. Flow cytometry with Annexin V (AV) and Propidium Iodide (PI) demonstrated that GBM1600 spheres infected with MELK shRNA have more cells in early (AV(+), PI(−)) and late (AV(+), PI(+)) stages of apoptosis, compared to the control shRNA infected cells (Fig. 2A). We then introduced MELK wild type or D150A cDNA into these infected cells. As a control, we used GFP-overexpression vector. When wildtype MELK was restored in MELK shRNA infected GBM1600 spheres, we observed a partial reversal of the effects on apoptosis (AV(+), PI(+) cells; 30.7% in the control samples vs. 15.4% in MELK wild type samples) [18]. In contrast, overexpression of MELK D150A failed to rescue the effects of MELK knockdown (AV(+); PI(+) cells; 37.0% in MELK D150A sample), indicating that this mutant MELK lacks the ability to recover MELK elimination-induced cell death. Given that our previous observation that the D150 residue of MELK is required for the interaction of MELK protein with the oncogenic transcriptional factors c-JUN and FOXM1 in a cancer-specific manner [18], [19], it is likely that the kinase domain is essential for MELK-driven GSC survival.

### Identification of C1 as an Inhibitor for Mitotic Kinases Including MELK

The above data raised a possibility that the kinase domain of MELK is a potential therapeutic target for GBM. We therefore sought to discover small molecules that specifically inhibit its kinase activity. To this end, we performed an *in silico* screening of small molecules and identified a benzo[e]pyridoindole, C1 (Fig. 2B), as a multi-kinase inhibitor with significant activity against the mitotic kinases, MELK and Aurora B. Effects of C1 on other kinases exhibited substantially lower potency [21]. Computer-based molecular structure analysis supported the predicted docking of C1 to the ATP-binding site of MELK protein (Fig. 2C). The inhibition of MELK kinase activity by C1 was further validated, as we found that compound C1 inhibited the kinase activity of recombinant MELK protein with an IC50 of 42 nM *in vitro* (Fig. 2D).

### C1 Treatment Inhibits GSCs to a Greater Extent than Non-GSCs *In vitro*

**Figure 3:**
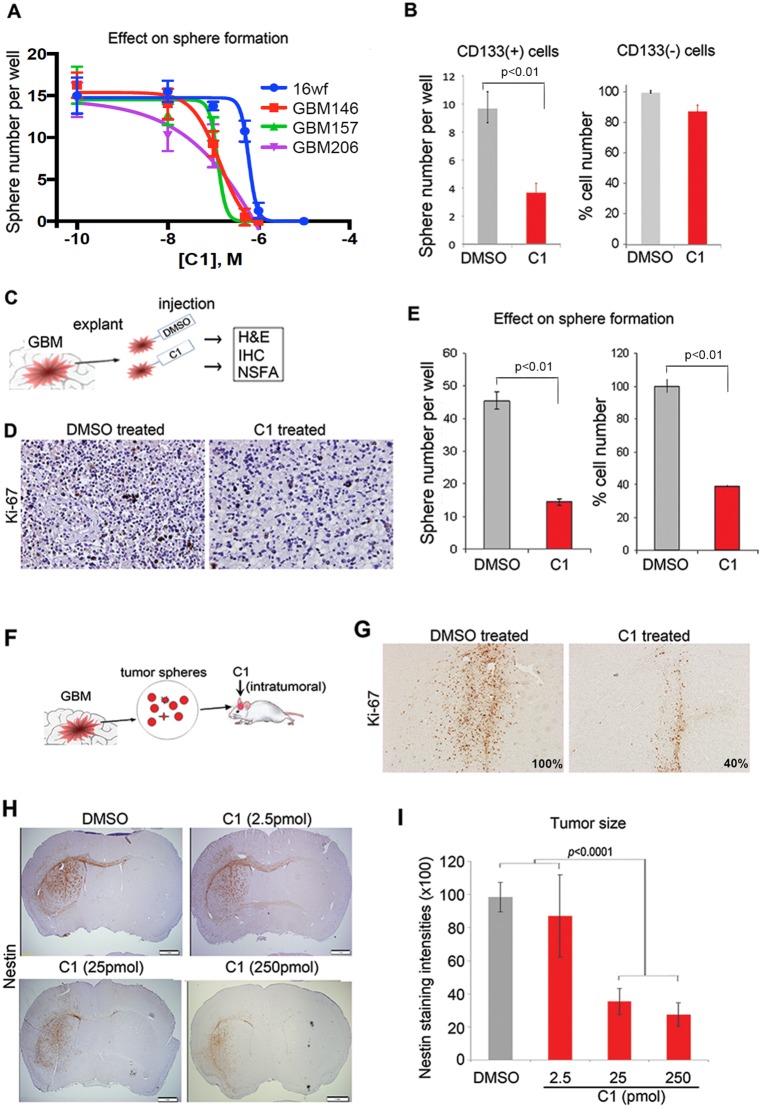
C1 treatment inhibits GSC proliferation *in vitro* and *in vivo*. A, Graph of neurosphere forming assay indicating the relative neurosphere numbers of C1-treated patient-derived GBM samples (GBM146, GBM157, and GBM206) and normal neural progenitors (16wf). B, CD133(+) and (−) cells, separated from GBM157-derived sphere cultures, were treated with 1 μM C1 or DMSO (control) under the identical serum-free conditions for 48 hours. The effect on CD133(+) cells was assessed by the neurosphere number per well, and the effect on CD133(−) cells was assessed by the % change of the total cell number in comparison to the control sample. C, Schematic showing organotypic slice cultures explanted GBM tissues and treated with C1 or DMSO (control) for 16 hours and evaluated with H&E, Ki67, and Nestin staining. D, Immunohistochemistry of C1- or DMSO-treated GBM slice cultures with anti-Ki-67 monoclonal antibody (Original magnification, ×200). E, Graph indicating the numbers of neurospheres (left) or total cells (right) in serum-free medium derived from C1- or DMSO-treated slice cultures for 16 hours. F, Schematic drawing of the effect of C1 treatment for the mouse intracranial GBM models derived from GSCs. Cells from GBM157 spheres were injected intracranially into immunocompromised mice (C1 mice: n = 4, control mice: n = 12). At day 7 post transplantation, C1 was injected intratumorally at quantities of 2.5 pmol (n = 3), 25 pmol (n = 4), or 250 pmol (n = 5). G, Representative images for immunohistochemistry with Ki-67 staining of GBM slice cultures treated with 25 pmol C1 or DMSO at day 10 of treatment. Ki-67 positive cells in each group were analyzed automated digital image analysis (Original magnification, ×200). H, Representative images for immunohistochemistry with human-specific Nestin antibody using GBM157-derived mouse intracranial tumors treated with varying doses of C1 or DMSO intratumoral injection (bar: 1 mm). I, Graph indicates tumor sizes in each group as determined by Nestin staining intensities analyzed using automated digital image analysis.

Next, we sought to assess the sensitivity of GSCs to C1 *in vitro*. First, we compared the effects of C1 treatment on neurosphere formation from patient-derived GBM cells and normal neural progenitors [17]. We incubated the 3 GSC samples (GBM146, GBM157, and GBM206) and normal neural progenitors (16wf) with varying concentrations of C1 to measure the impact on neurosphere formation. C1 treatment attenuated neurosphere formation of all 3 GBM samples at substantially lower doses (GBM146: 440 nM; GBM157: 370 nM; GBM206: 370 nM) than normal progenitors (16wf: 790 nM)(Fig. 3A).

We then performed FACS analysis with GSCs treated with either C1 or DMSO, as the expression of the cell surface CD133 is well-recognized as a surrogate, but not definitive, marker for GSCs [24], [29], [30]. Following separation of GBM157 cells into CD133(+) and CD133(−) populations by cell sorter, individual cell populations were separately treated with 1μM of C1 or DMSO under the identical serum-free conditions for 48 hours. CD133(+) tumor cells with C1 treatment showed significant reduction of the sphere number compared with the DMSO-treated cells. On the contrary this C1-induced cell number reduction was not apparently observed in CD133(−) tumor cells (p<0.01; Fig. 3B). Collectively, C1 has more potent inhibitory effect on GSC growth compared to the growth of non-GSCs or normal progenitors *in vitro*.

### C1 Induces Apoptotic Cell Death in Organotypic Slice Cultures of GBM Surgical Specimens

Organotypic slice culture of tumor tissues is one experimental model readily available to assess the influence of drug treatment on tumor cells *in situ* without significantly destroying the tumor microenvironment [31]. We performed intratumoral injection of C1 or DMSO using 2 patient-derived slice cultures of GBM tumors (Fig. 3C). Immunohistochemistry for Ki-67 exhibited the presence of abundant proliferating tumor cells in the DMSO treated samples but not in the C1 treated ones (*p*<0.0026; Fig. 3D), suggesting that C1 treatment suppressed GBM cell proliferation *in situ*. We then assessed the effect of C1 injection on sphere-forming GSCs within these slice cultures. After the C1-treated or DMSO-treated slice cultures were dissociated into single cells, we performed neurosphere formation assay. As shown in Fig. 3E, we found the significant reduction both of the number of neurospheres and the total cell numbers by C1 treatment in both cases (*p*<0.01). These data suggest that C1 has a potent inhibitory effect on the GBM cell proliferation and GSC growth under the condition of a relatively preserved microenvironment for GBM tumors.

### C1 Inhibits GBM Cell Growth *In vivo*

Given the potent inhibitory effects of C1 on GSCs *in vitro* and *ex vivo*, we next investigated the effect of C1 treatment on mouse models of GBM *in vivo*. Following transplantation of GBM157 cells into immunocompromised mouse brains, we injected C1 at day 7 with quantities of 2.5 pmol, 25 pmol, and 250 pmol into the tumor cavities (Fig. 3F). At day 3 post-C1 injection, we observed a substantial decrease in proliferating GBM157 cells, as evidenced by far fewer cells labeled with Ki-67(+) in C1-treated (25 pmol and 250 pmol) tumors than DMSO-treated (40% in DMSO-treated tumors vs. 32% in C1-treated tumors (25 pmol); Fig. 3G).

With the other set of mice harboring GBM157-derived intracranial tumors, we measured tumor sizes at 8 weeks post-transplantation. While there was only a marginal difference in tumor sizes between the control and C1 treatment at 2.5 pmol, tumors treated with C1 at both 25 pmol and 250 pmol exhibited a 3-fold decrease in size compared to the control (n = 7, *p*<0.0001; Fig. 3H and I). These results suggest that intra-tumoral treatment with C1 diminishes the *in vivo* growth of GSC-derived tumors in mouse brains.

### C1 Treatment Induces Early Mitotic Exit and Subsequent Mitotic Catastrophe and Cell Death of GSCs

**Figure 4:**
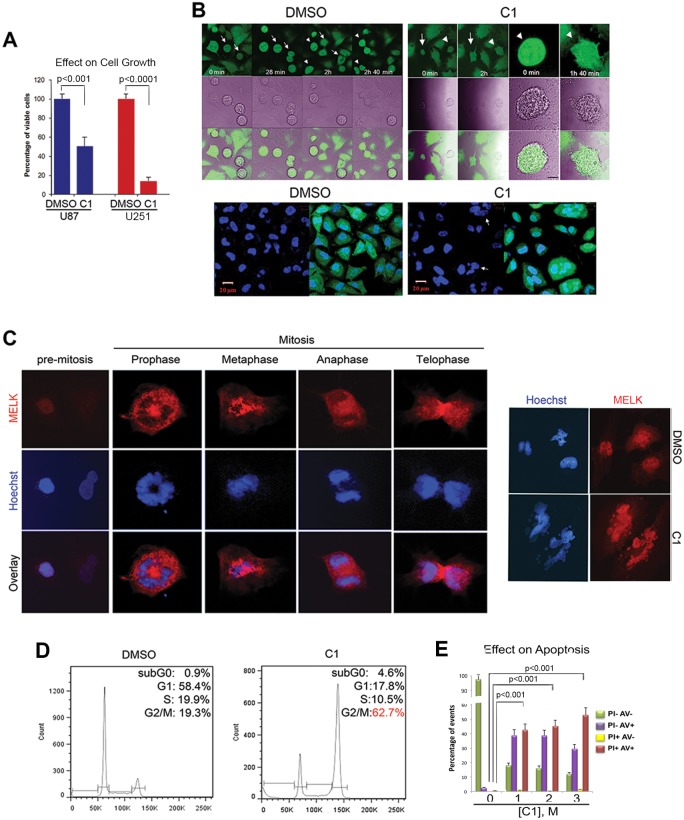
C1 treatment accumulates GSCs in G2/M and triggers subsequent mitotic catastrophe. A, Proliferation assays on two glioblastoma cell lines (U87 and U251). U87 and U251 cells were treated with 5.7 μM C1 or DMSO. Cells were trypsinized and estimated by counting, in duplicate, after 72 h of treatment. Two different experiments were conducted with similar results. B, Time-Lapse on mitotic U251 cells stably expressing GFP was performed in the absence (DMSO) or in the presence of C1 (at 1 μM). The compound was added to the cell culture just before imaging and then cells were continuously imaged. Three independent experiments were conducted and 10 to 15 fields were followed in each. None of the followed mitotic cells divided in two daughter cells. Representative field is imaged, DNA is in blue and the merge shows GFP and DNA. Several polyploid cells were present in the image. Arrows and arrowheads in upper panels of DMSO and C1- treated cell indicate the same cells through time-lapse. The elapse times are indicated on each photo, in some assays, a zoom of one cell is shown (the red bar represents 5 μm) this cell is present on the former field and labelled with an arrow). Images in bottom panels show DNA only (left) and DNA overlap with GFP (right) after 72-hour treatment with DMSO or C1 (the red bars on each panel represent 20 μm). Arrows in the bottom panel of C1-treated cells indicate mitotic catastrophe by C1 treatment. C, Pictures demonstrate pre-mitotic phase (left panel), mitotic (mid panel) process by full karyokinesis and cytokinesis and after cell division (right panel). MELK expression was determined with immunocytochemistry of GBM1600 cells with anti-MELK antibody (red), chromatin staining with Hoechst stain (blue). Picture of pre-mitosis shows GBM1600 cells highly expressed MELK at pre-mitosis phase (400× magnification). Then GBM1600 cells were treated with 5 μM C1 or control and were subjected to immunocytochemistry 3 days later with anti-MELK and chromatin staining (640× magnification). Data were confirmed by three independent experiments. C1 treated cells are micronucleated at metaphase and followed multinuclear chromatin condensation (mid panel). Right panel show multinuclear asymmetric divided chromatin of C1 treated cell compared with DMSO treated cell. D, Flow cytometric analysis of C1- and DMSO-treated GBM1600 cells with Propidium Iodide at 3 days after treatment shows 62.7% of C1-treated cells resulted in the G2/M arrest, whereas the control cells have 19.3% of the G2/M arrested cells.E, Graph indicating the proportions of live, early apoptotic, and late apoptotic U251 cells with varying doses of C1 or DMSO.

Next, we investigated the mechanism of cell cycle arrest induced by MELK kinase inhibition in 2 GBM-derived cell lines, U87 and U251. We first confirmed that C1 decreases their growth *in vitro*. With both cell lines, the total viable cells were significantly decreased by C1 treatment compared to DMSO-treated cells (U87: *p*<0.001, U251: *p*<0.0001; Fig. 4A). We then performed time-lapsed immunocytochemistry with C1-treated U251 cells expressing GFP. The top panel in Fig. 4B (left side is treated with DMSO, right side with C1) displayed mitotic cells readily dividing and giving rise to two daughter cells (arrows and arrowheads). C1-treated mitotic U251 cells could not undergo anaphase and prematurely escaped from mitosis, as evidenced by the round cell spread on the substrate at 1 hour 40 min (cell focused from former field) post-treatment. As showed in the bottom panel of Fig. 4B, C1-treated cells at 72 hours formed polyploid cells with multiple abnormal micronuclei within single cells, unlike the control cells exhibiting normal, euploid nuclei. This phenomenon is known as “*mitotic catastrophe*,” which irreversibly drives cells into apoptosis, necrosis, or senescence [32]. In contrast, when U251 cells were treated with temozolomide (TMZ), the current first-line chemotherapy for GBM, cell growth was attenuated without affecting mitotic division (data not shown). Collectively these data suggest that the mechanism of cell death by C1 is distinct from that of TMZ.

Since C1 is a multi-kinase inhibitor, we then sought to determine the major molecular target of C1 in GSCs. We postulated that if C1 attenuates GSC survival mainly through MELK kinase inhibition, the phenotype of C1-treated GSCs should be identical to that of shRNA-mediated MELK elimination. Previously, we found that MELK knockdown prevents GSCs from proper mitotic progression, resulting in accumulation of tumor cells in the G2/M phase, generation of polyploid cells with multiple micronuclei, and subsequent apoptotic cell death [17], [33]. We monitored C1-treated GBM1600 cells from pre-mitosis phase until mitotic completion. As expected, GBM1600 cells at pre-mitosis phase express high levels of MELK protein (Fig. 4C, left panel). We then followed the effects of C1 through karyokinesis and cytokinesis of GBM1600 cells. These C1 treated GBM1600 cells demonstrated micronucleated, chromatin condensation (signs of apoptosis) and asymmetric division (Fig. 4C, middle and right panels). DMSO-treated cells, on the other hand, showed round chromatin and homogenous cell size and figures. To support these data, cell cycle analysis with PI showed the accumulation of C1-treated GBM1600 GSCs at the G2/M phase (62.7% with C1 treatment vs. 19.3% with DMSO treatment; Fig. 4D). To determine the subsequent cell fate of C1 treated cells, we performed FACS analysis with U251 cells using Annexin V and PI (Fig. 4E). Both early and late apoptotic fractions were significantly increased in C1-treated cells in a dose-dependent manner (*p*<0.001; Fig. 4E). Taken together, these data indicate that C1 treatment induces failure of mitotic progression to undergo anaphase and G2/M arrest of GSCs. These effects subsequently promote early mitotic exit, metaphase-associated mitotic catastrophe, and subsequent apoptotic cell death.

### C1 Sensitizes GSCs to Radiation Treatment

**Figure 5:**
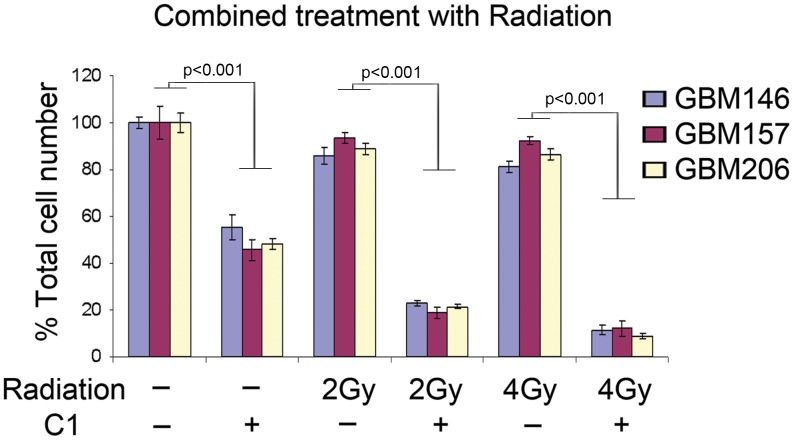
C1 sensitizes GBM cells to radiation-induced cell death, *in vitro*. Graph indicating the relative total cell numbers after monotherapy (1 μM C1 or radiation) or after combination therapy. Cells treated with combination therapy were radiated (2Gy or 4Gy) at 24 hours post-C1 treatment and the subsequent total cell numbers were counted at 3 days after irradiation. Data are shown from 3 separate experiments.

Irradiation is the current first line post-surgical therapy for GBM patients. Nonetheless, the survival benefit for GBM patients of radiation treatment is no greater than 3 months [34], [35]. One potential reason for the limited efficacy of this treatment is the rapid induction of the DNA damage repair genes and proteins in tumor cells [4]. In particular, GSCs are known to upregulate these DNA repair genes more efficiently than non-GSCs, which may partially explain their pronounced radioresistance [4], [36]. Since we found that inhibition of MELK-mediated pathway potently suppressed the DNA damage repair pathway in GSCs (Fig. 1D), we hypothesized that C1 treatment combined with radiation would have a greater efficacy over radiation alone. To address this question, we used radiation treatment at sub-lethal doses (2Gy and 4Gy) for GSCs with or without C1 treatment (Fig. 5). While radiation alone did not noticeably affect GSC survival at the indicated doses, the combination with 1μM of C1 treatment resulted in a significant reduction in the GSC growth (*p*<0.0001), indicating that C1 treatment sensitizes GSCs to radiation-induced cell death *in vitro*.

## Discussion

In this study, we demonstrated that MELK acts on GSC survival through its kinase activity. We performed the computational structure analysis of MELK protein to determine the ATP binding region of this kinase. Using this information, we identified C1 as a kinase inhibitor with substantial potency against MELK. C1 treatment not only induced G2/M arrest and subsequent mitotic catastrophe of GSCs *in vitro,* but also inhibited the growth of GSC-derived tumors *in vivo*. Finally, we found that C1 treatment sensitizes GSCs to radiation-induced cell death, supporting C1 as an attractive molecule-targeting therapy to combine with current standard protocols of GBM treatment.

Our study contributes several novel findings to the current understanding of the GBM pathophysiology. To our knowledge, we present the first pre-clinical data for MELK kinase inhibition-mediated suppression of GBM cell growth *in vivo* using a small molecule kinase inhibitor. Previously, we found that the oxo-group of C1 is necessary for kinase inhibitory activity [27]. Our *in silico* model predicted that the oxo-group of C1 forms a strong hydrogen bond with the Tyr88 residue of the ATP-binding triplet domain, confirming our earlier report [21]. Our study therefore illustrates that pharmacological inhibition of kinase activity at the ATP binding site has very attractive therapeutic potential and strongly warrants further study and drug development. However, similarity of the structure of the ATP binding site has been noticed with various protein kinases. In fact, our prior study exhibited that C1 inhibits multiple protein kinases with potent effects on not only MELK but also Aurora kinases and Chk2 with the highest affinities towards Aurora B and MELK [21]. With the current dataset, we cannot draw a definitive conclusion that the potent effect on GSCs by C1 treatment is solely due to MELK inhibition. Both Aurora B and MELK are mitotic kinases, and the relative inhibition of each by C1 is indistinguishable. However, the selectivity towards CD133(+) cells in this study may suggest that MELK inhibition predominantly contributes to C1 efficacy in GSCs [10], [16]. The individual effects of C1 on these kinases aside, we found that inhibition of the MELK-mediated pathway also suppresses expression of Aurora kinases (Fig. 1B and C). In addition, we recently revealed that the oncogenic transcription factor FOXM1 is a substrate for MELK specifically in GSCs. Given that Aurora kinases are known downstream targets of FOXM1, multi-kinase inhibition would provide better efficacy with lower doses to avoid unwanted toxic effects on normal orgains [19]. Further work is definitely needed to determine the contributions of Aurora kinases and other protein kinases to GBM pathogenesis and to clarify the full scope of C1 target molecules.

Given that mutations in the kinase domain or in other molecular elements of MELK do not appear to be a frequent event in cancers, the distinct function of MELK in normal and oncogenic stem cells is likely to be epigenetic. Several recent studies, including our own, have implicated MELK in cell cycle regulation [37], [38], successful cell division [39], and suppression of apoptosis [16], [40], [41], making it entirely possible that MELK contributes to tumor initiation and propagation through molecular interactions with deregulated oncogenes and/or tumor suppressor genes. In support of this notion, we recently demonstrated that a JNK pathway-driven interaction of MELK with another transcription factor/oncoprotein c-JUN is essential for GSC survival, proliferation, and radioresistance in a p53 dependent manner [18]. Introducing a point mutation in MELK protein at the D150 residue, which is required for proper kinase activity [28], attenuated the protein complex formation with c-JUN. Furthermore, this interaction with c-JUN was unique to GSCs and was not found in normal neural progenitors. Collectively, it is possible that C1 interrupts the oncogenic JNK signaling cascade through inhibition of MELK kinase activity and the resulting interaction with c-JUN. Given that JNK signaling orchestrates a variety of cellular processes, pharmacological inhibition of MELK, a more downstream and possibly cancer-specific protein, may lead to fewer off-target effects and greater specificity in targeting cancer cells. Further studies are required to elucidate this possibility.

The potent radioresistance of GSCs has been partly attributed to upregulation of the ATM/ATR DNA damage response pathway [42], [43]. In this study, we found that the greatest effect of MELK signaling inhibition was on the ATM/ATR DNA damage response pathway and C1 treatment radiosensitizes GBM cells at least *in vitro*. Recently, Golding *et al*. reported that ATM inhibition effectively radiosensitizes GBM cells without harming normal neural progenitor cells [44]. Further, Raso *et al.* demonstrated that radiosensization through ATM inhibition occurs preferentially in GSCs but not in non-GSCs [45]. We previously demonstrated that treatment of GSCs with Siomycin A reduces GSC-derived tumor growth *in vivo* without causing a noticeable harmful effect on normal brain cells [16]. Taken together, MELK inhibition may attenuate radiation-induced ATM/ATR activation in GSCs that are essential for their role in the DNA damage repair and survival.

Regarding the clinical application of C1 for GBM therapeutics, some open questions remain. In fact, the efficacy of chemotherapy of brain malignancies is often hampered by the presence of the blood-brain barrier (BBB). From the point of molecular weight, the size calculated from the structure of C1 is 293 Da, which is presumably small enough to penetrate the BBB. However, the permeability of the BBB is not solely dependent on the molecular size but also affected by several kinds of drug property and circumstances. Given the potent effect of C1 treatment on mouse GBM-like tumor models *in vivo*, it is attempted to evaluate the permeability of the BBB and bioavailablity/stability of C1 *in vivo*.

In conclusion, our data indicate that C1 is a novel inhibitor for protein kinases with substantial inhibitory effect on MELK. This study suggests that pharmacological inhibition of MELK kinase activity represents an attractive therapeutic approach for GBM that may overcome the resistance seen after current, standard treatment protocols. We postulate that C1 may also effectively treat a variety of cancers with elevated activation of MELK.
